# Vaginal microecological characteristics of women in different physiological and pathological period

**DOI:** 10.3389/fcimb.2022.959793

**Published:** 2022-07-22

**Authors:** Liping Shen, Wei Zhang, Yi Yuan, Weipei Zhu, Anquan Shang

**Affiliations:** ^1^ Department of Obstetrics and Gynecology, Changning Maternity & Infant Health Hospital, Shanghai, China; ^2^ Department of Obstetrics and Gynecology, The Second Affiliated Hospital of Soochow University, Suzhou, China; ^3^ Department of Laboratory Medicine, Shanghai Tongji Hospital, School of Medicine, Tongji University, Shanghai, China; ^4^ Department of Laboratory Medicine, Jiaozuo Fifth People’s Hospital, Jiaozuo, China

**Keywords:** vaginal microecological, vaginal microbiota, reproductive health, inflammation, cancer

## Abstract

The vaginal microbiota, the host endocrine system, the vaginal anatomy, and the local mucosal immunity comprise the vaginal microbiota, which interacts with each other to maintain the balance of the vaginal microbiota, which maintains female reproductive health. Puberty, menstruation, pregnancy, and menopause are four phases women go through during their reproductive and post-reproductive years. Vaginal microbiota composition and abundance are heavily influenced by estrogen and progesterone, which start at puberty and continue during the reproductive years in a dynamic balance with some fluctuations. Estrogen promotes proliferation of vaginal epithelial cells and increases glycogen storage, while progesterone lyses vaginal epithelial cells, facilitating the release of glycogen to maintain normal pH. This review summarizes the latest national and international evidence on the composition and distribution of vaginal microecology in women during different physiological and pathological periods and proposes a hormone-driven microbial diversity hypothesis to explain the temporal patterns of vaginal microbial diversity during the female reproductive cycle and menopause. A relatively balanced vaginal microecological system has a positive effect on the maintenance of female health. An imbalance in the ratio of flora can lead to susceptibility to infections or reproductive complications. The study of human microecology and its role in the development and progression of human disease is essential for the prevention, diagnosis, and treatment of related obstetric and gynecologic conditions.

## Introduction

Microorganisms (including bacteria, archaea, protozoa, fungi, and viruses) exist in trillions in the human body (referred to as the “microbiome) (Zhang et al., 2018). These microorganisms are responsible for regulating food metabolism and maintaining the integrity of the intestinal epithelial mucosa. In the presence of an imbalance in these microorganisms, various diseases can occur. In addition to maintaining the structural integrity of the intestinal epithelial mucosa and regulating food metabolism, imbalances in intestinal flora are associated with a variety of diseases. The intestinal flora plays a critical role in helping the host absorb food, develop the immune system, and protect against infection, as it is involved in all aspects of human physiology and metabolism (Wang et al., 2015; Sepich-Poore et al., 2021). In recent years, the vaginal microbiota has received much attention. This is especially true with the development of modern sequencing technologies. As a result, our knowledge of the composition of the microflora that hosts the human vaginal environment as well as its potential applications has increased. The bacteria found in the vaginal cavity are quite varied. The beginning of the development and expansion of the vaginal microbiome occurs at birth, and variations in its composition are primarily caused by a variety of genetic, dietary, and environmental variables. Depending on the types and activity of microorganisms in the vaginal environment, vaginal microecology may cause varying metabolic and immunological responses. Alterations in the microecological balance of the human vaginal tract can result in dysbiosis, inappropriate inflammatory responses, and abnormal immune responses, all of which can contribute to a wide range of female reproductive health problems.

**Figure 1:**
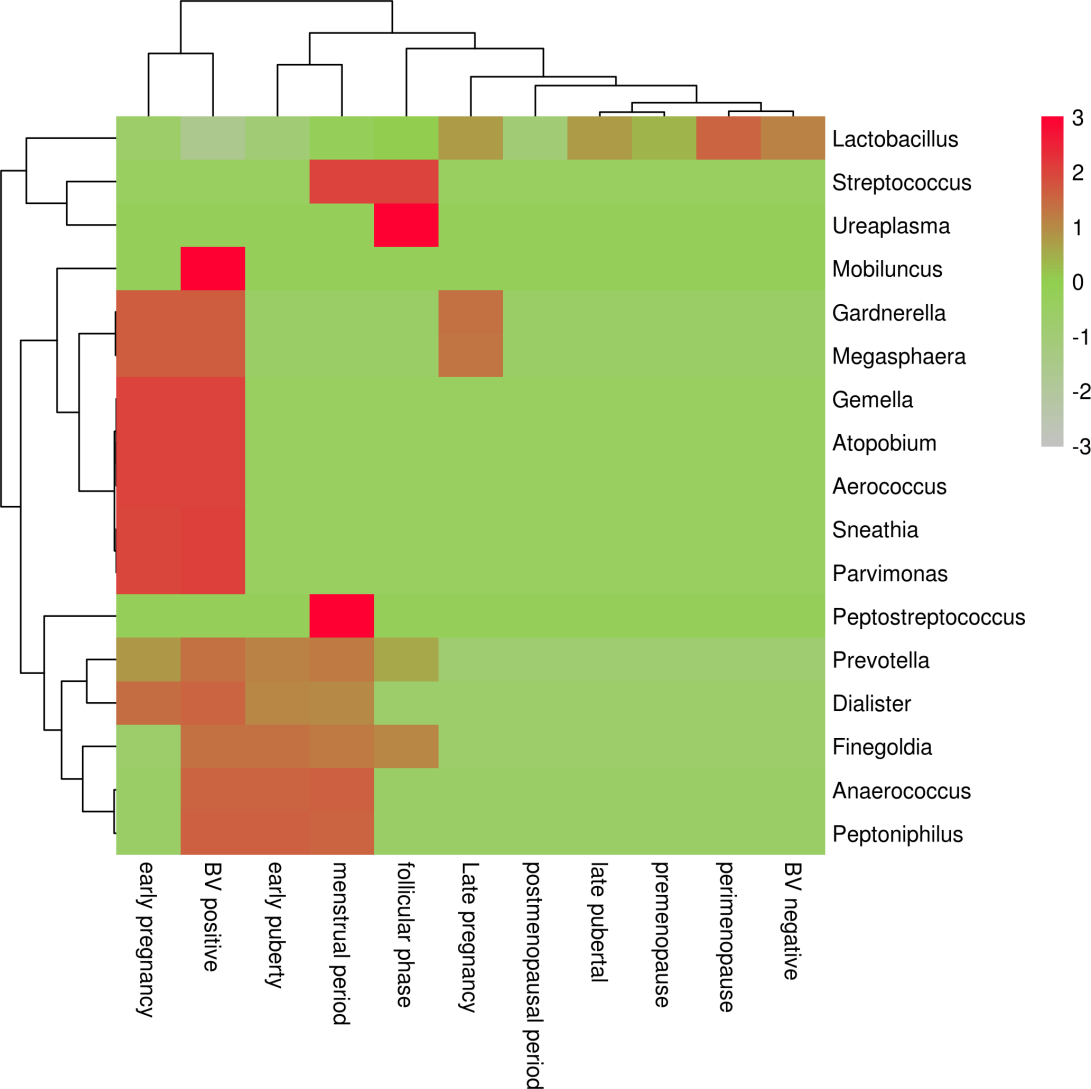
Heat map of vaginal microflora distribution in women by period.

Ravel et al. (Ravel et al., 2011) 2011 conducted a study in which the vaginal bacterial communities of 396 asymptomatic North American women representing four races (white, black, Hispanic, and Asian) were sampled and characterized by high-phosphate sequencing of the 16S rRNA gene to identify microbial species. The study identified five types of vaginal bacterial communities, termed community state type (CST), in women of childbearing age. He pointed out that it is completely normal for women to experience a change from one CST to another within a short period of time. Each CST has a dominant Lactobacillus: CST type I (with Lactobacillus curvatus as the dominant bacterium), CST type II (with Lactobacillus garciae as the dominant bacterium), CST type III (with Lactobacillus inertus as the dominant bacterium), CST type IV (with other various bacteria such as anaerobes as the dominant bacterium), and CST type V (with Lactobacillus janus as the dominant bacterium). Among them, CST type IV was divided into type IVA (BVAB1 as dominant bacterium), type IVB (Gardnerella vaginalis as dominant bacterium), type IVC0 (Prevotella as dominant bacterium), type IVC1 (Streptococcus as dominant bacterium), type IVC2 (Enterococcus as dominant bacterium), type IVC3 (Bifidobacterium as dominant bacterium) and type IVC4 (Staphylococcus as dominant bacterium) (**Figure 1**).

In most women, the vaginal microbiota is dominated by lactic acid-producing strains of the genus Lactobacillus. In addition to lactic acid, other substances such as hydrogen peroxide and bacteriocins are produced by lactobacilli in the vaginal microenvironment to inhibit the growth of potential pathogens. Women typically go through four distinct phases during their reproductive and late reproductive years, namely puberty, menstruation, pregnancy and menopause. The composition and abundance of the vaginal microbiota is primarily influenced by estrogen and progesterone, an effect that begins at puberty and continues throughout the reproductive years in a dynamic balance with slight fluctuations (Gajer et al., 2012). Estrogen promotes proliferation of vaginal epithelial cells and increases glycogen storage, while progesterone lyses vaginal epithelial cells, facilitating glycogen release.

## Micro-ecological characteristics of the female reproductive tract during the physiological period

Understanding the establishment of the human microbial community is essential for understanding human development and physiology. It has been suggested that newborns exhibit a complex microbial community in the gut during the first week of life, with dynamic fluctuations in bacterial composition continuing until a relatively mature equilibrium is reached around 1-3 years of age (Mackie et al., 1999). During the first week of life, the intact neonatal gut microbiota is dominated by Actinobacteria (including Bifidobacterium), Proteobacteria, Bacteroides and Firmicutes (including Lactobacillus, which dominates the vaginal flora). In contrast, newborns weighing < 1200 g were dominated by Firmicutes and Prevotella, with a much smaller predominance of Actinobacteria (Stout et al., 2013). These studies raise the possibility that microbial colonization in infants originates at the level of the maternal-fetal interface and that the colonization of such microorganisms may vary with the length of gestation. As the newborn is delivered from the mother, the microorganisms in the body change along with the growth and development of the child. The microecological characteristics of the female reproductive system during these periods are unknown because of the limited collection of samples from the reproductive tract of infants and children.

### Puberty

In several international research cohorts, the vaginal microbiota has been found to be dominated by taxa belonging to five bacterial phyla, namely Anaplasma, Actinomycetes, Thick-walled Bacteria, and Clostridium (Kaur et al., 2020). Adolescence is a period of rapid growth as females transition from childhood to sexual maturity, and is a process of gradual endocrine, reproductive, physical, and psychological maturation. In early adolescence, when estrogen and progesterone levels are low, the vaginal microbiota is mainly rich in Aspergillus, Bacteria and Actinobacteria, and has relatively high alpha diversity (richness, uniformity, proportional diversity) (Kohlberger and Bancher-Todesca, 2007), rich in Prevotella, Bacteroides, Gastrodia, Anaerobes, small classes of Bacteroides and Lactobacillus; with maturation of the gonads and entry into late adolescence, the vaginal microbiota becomes dominated only by thick-walled bacteria. In addition, the level of sex hormones gradually increases, leading to thickening of the vaginal epithelium and glycogen storage, which provides an adequate source of nutrients for the growth and proliferation of vaginal microorganisms. Distribution (Stapleton, 2016; Wessels et al., 2018).

### Menstrual period

In the female physiological state, the luteal phase (before to the commencement of menstruation) is the peak of sex hormones in the female body, accompanied with thickening of the endometrium and vaginal epithelial cells and glycogen accumulation in preparation for prospective embryo implantation. When the level of sex hormones abruptly drops due to infertility, the endometrium separates from the uterine wall and the vaginal epithelial cells are shed, accompanied by the menstrual blood flowing out of the vagina. At this time, the vaginal environment is rich in blood cells and epithelial cells, and a new cycle of menstruation begins. The vaginal environment is a breeding ground for numerous microorganisms, including Clostridium, Aspergillus, Aspergillus, and Actinobacteria; the taxonomic composition of the vaginal microbiota during menstruation is similar to that of early adolescence, but studies have identified two genera of bacteria that are unique to this period: Streptococcus digestiveis and Streptococcus. In normal conditions, the pH of menstrual blood is comparable to that of normal blood (7.2–7.4), and an increase in vaginal pH leads to an increase in the number of anaerobic microorganisms, which typically exist as symbionts (Eschenbach et al., 2000). However, in an environment with a neutral pH, lactic acid produced by lactobacilli has no protective antibacterial effect (Morison et al., 2005). Consequently, during menstruation, the interaction of menstrual blood with the vagina neutralizes the acidic vaginal milieu, and the rise in vaginal PH results in a significant increase in the number of anaerobic microbes that serve as symbionts. In addition, during menstruation, iron in iron-containing heme from the damaged blood cells readily becomes the primary source of nourishment for a number of bacteria (Roberts et al., 2019). Vaginal microorganisms such as Streptococcus and Gardnerella secrete iron chelator complexes to obtain iron deposited on the vaginal mucosal surface (Scholl et al., 2016). In the human vaginal local immune system, neutrophil gelatinase-associated lipocalin (NGAL) inhibits the growth of iron-dependent bacteria by preventing iron storage (Witkin and Linhares, 2017). In vaginal microecosystems dominated by Lactobacillus, intravaginal NGAL levels are greater. During menstruation, menstrual blood produces a rise in vaginal pH and iron, as well as a drop in the quantity of Lactobacillus, resulting in an increase in vaginal microbial diversity (Kaur et al., 2020). With the transition from the menstrual phase to the follicular phase, sex hormones begin to rise gradually and the epithelial cells of the vaginal wall thicken and secrete more glycogen, which degrades to produce lactic acid and hydrogen peroxide, lowering the vaginal pH and favoring the proliferation of Lactobacillus while decreasing the number and diversity of other anaerobic bacteria (Onderdonk et al., 2016).

### Pregnancy period

During pregnancy, the female body experiences significant metabolic, immunological, and endocrine changes (Soma-Pillay et al., 2016). Pregnancy period is a natural physiological process during which a woman enters the reproductive phase. Alterations are made concurrently to the structure, composition, and abundance of microbial communities that colonize various bodily areas. 2019 Ceccarani et al. (Ceccarani et al., 2019)samples from a pregnancy cohort demonstrated an increase in the relative abundance of the thick-walled phylum with increasing gestational weeks; in addition, an entirely new group of microbiota was observed in the vaginal microenvironment in early pregnancy (Atopobium, Aerococcus, Gemella, Sneathia, Parvimonas, Gardnerella, and Megasphaera). By the middle of pregnancy, the quantity of this new vaginal microbiota declines dramatically and is replaced by an increase in the abundance of Lactobacillus. Existing studies have determined that the diversity of the vaginal microbiota is a significant predictor of pregnancy outcome, with a significant increase in the diversity of vaginal microbes in women with preterm birth compared to women with gestational age; in addition to microbial diversity as a potential indicator of impending preterm birth outcome, recent studies have also demonstrated that “species level” fluctuations in the vaginal microbiota are associated with preterm birth. Recent studies have shown that fluctuations in the “species level” of the vaginal microbiota (especially regarding Lactobacillus) are associated with preterm birth, such as Stimia, TM7-H1 and BVA-B1 (bacterial vaginosis-associated bacteria) (Fettweis et al., 2019), making the dynamic composition and functional differences in vaginal microbes a current hot topic of research on preterm birth. There are diverse alterations in the oral, placental, intestinal, and vaginal microbiota of pregnant women at various stages. When the vaginal flora is abnormal due to other factors, it can cause adverse maternal, fetal, and neonatal outcomes, such as: preterm miscarriage, spontaneous abortion, fetal arrest, preterm labor, spontaneous preterm labor, gestational diabetes, preeclampsia, premature rupture of membranes, preterm labor, spontaneous preterm labor, gestational diabetes, preeclampsia, premature rupture of membranes, preterm labor, spontaneous preterm. In order to protect the growing fetus and adapt to dynamic changes in physiological functions, the immune system of the body undergoes adaptive regulation. Pregnancy requires immunological resistance for human reproduction because a fetus must be obtained from two genetically distinct parents. The egg, a mother cell, has maternal antigens that the immune system identifies as “self.” When the sperm enters the egg in the fallopian tube, it causes several changes that create maternal and paternal antigens. As long as the embryo has no foreign antigens, it is a maternal cell. Once the embryo presents its own antigens, it is defended from maternal immune cells by the zona pellucida. The maternal oocytes provide early protection. The embryo interacts with the maternal system after a few days. When a donor embryo is successfully implanted using assisted reproduction methods, it must communicate with the mother’s immune system to avoid being attacked. 4–5 days after embryo transfer they communicate with each other. During this period, endometrial initiation, immunological tolerance and implantation occur (Bardos et al., 2019). In the early stages of pregnancy, the immune system weakens to facilitate implantation of the embryo, the variety of vaginal flora grows, and the amount of Lactobacillus drops. In turn, this allows Lactobacillus lactis to colonize and multiply in the vagina, lowering the vagina’s pH and limiting the colonization and growth of other bacteria. This kind of stable vaginal flora in late pregnancy inhibits the growth of harmful bacteria in the reproductive tract and reduces the incidence of reproductive tract infections, hence promoting the healthy development of the gestational sac or fetus. During mid- and late-pregnancy, the body’s autoimmune process starts to re-establish itself, and the immunological capacity steadily develops. Throughout pregnancy, this “reconnection of the immune system” induces a low-grade inflammatory response on the intestinal, vaginal, oral, and placental mucosal surfaces. This leads in alterations to the structural composition and abundance of the microbial communities inhabiting these various bodily areas (Koren et al., 2012).

### Climacteric

In contrast to pregnancy, women in menopause (around 50 years of age) undergo significant changes in reproductive hormones, including reduced estrogen and increased follicle stimulating hormone levels (Xu et al., 2020). Premenopausal and perimenopausal women had a substantially same microbial makeup and a more stable zonation than postmenopausal women. The premenopausal and perimenopausal stages are dominated by the thick-walled phylum, while the postmenopausal stage is dominated by Aspergillus, Anaplasma, and Actinobacteria. Gliniewicz et al. (Gliniewicz et al., 2019) studied the vaginal microbiome of premenopausal and postmenopausal women, taking hormone replacement treatment (HRT) into account, in 2019. By measuring 16s rRNA gene copies, the researchers determined that postmenopausal women who underwent hormone replacement treatment had similar bacterial counts to premenopausal women. In contrast, the bacterial counts in postmenopausal women who did not undergo hormone replacement treatment were almost ten times lower (p<0.05) than in the other two groups (i.e., premenopausal and postmenopausal women who received hormone replacement therapy). In postmenopausal women, a rise in the amount of anaerobic bacteria (e.g., Bacteroides mimicus, Mobiluncus motile curvilinearis) and vaginosis-related bacteria, such as Gardia vaginalis, has been found in several investigations (Brotman et al., 2018). Shardell et al. (Shardell et al., 2021) performed a two-year cohort research in which premenopausal, perimenopausal, and postmenopausal women participated. Nearly fifty percent of postmenopausal women had low Lactobacillus levels in their vaginal microbiome. In addition, the low lactobacilli community had a greater frequency of reduced libido and vaginal dryness than the high lactobacilli population.

## Microecological characteristics of the female reproductive tract at the pathological stage

### Inflammatory phase

The female genital tract system can be divided into the upper genital tract (uterus and cervical canal), which is covered by a single layer of columnar epithelium, and the lower genital tract (vagina and vaginal part of the cervix), which is covered by multiple layers of squamous epithelium (Zhou et al., 2018). The epithelial cells on the surface of the genital tract mucosa interact with the underlying basement membrane to regulate their physiological differentiation. The vaginal health of women plays a key role in inflammatory diseases of the reproductive tract. Bacteria can infect other reproductive organs through the vagina, leading to disorders of the female reproductive system. An imbalance in the ratio of bacteria in the vagina is thought to contribute to the susceptibility of the reproductive organs to infection or complications.

In the female vagina, imbalances in the microbial community are thought to cause symptoms associated with bacterial vaginosis. BV is the most common cause of vaginal discharge and malodor, and is the most common vaginal syndrome in women of childbearing age. It is a vaginal infection caused by a decrease or absence of normal hydrogen peroxide-producing lactobacilli in the vagina and an increase in parthenogenic anaerobic and anaerobic bacteria. Earlier studies have reported that vaginal microorganisms in healthy women are predominantly dominated by thick-walled bacterial phylum. The enrichment of the thick-walled phylum was also observed in samples analyzed from Ceccarani’s BV-negative cohort, while in the BV intermediate and BV-positive cohorts the abundance of thick-walled bacteria decreased and was replaced by a higher relative abundance of Clostridium, Lactobacillus, Actinomyces, and Aspergillus phylum. The disruption of the vaginal micropores is thought to be the main cause of the altered vaginal environment and associated clinical symptoms (Muzny and Schwebke, 2016). The vaginal flora of healthy women is dominated by Lactobacillus, which inhibits the growth of pathogenic microorganisms by producing lactic acid and releasing various antimicrobial components such as cytokines, surfactants, and H202. The vaginal flora of BV-positive women is characterized by a decrease or absence of Lactobacillus and a high diversity of microbial complexes dominated by Gardnerella and Atopobium spp (Ravel et al., 2011). Pathogens invade the normal, healthy microbiota through multiple pathways by disrupting host physiology, consuming vaginal nutrients, disrupting the vaginal barrier through hydrolytic enzymes (e.g., sialic acidase, prolidase), and promotes the release of inflammatory chemokines and cytokines (IL-6, IL-8, IL-1, INF-α, etc.) (Onderdonk et al., 2016). Depletion of Lactobacillus spp. prevents vaginal pH from being maintained in the normal range (i.e., 3.8–4.5), and when some of these pathogens have the ability to form biofilms, they can cause a cascade of negative events in the host, such as persistent infections caused by a mixture of difficult-to-treat pathogens (Jung et al., 2017). A bacterial biofilm is a specific bacterial community structure formed by bacteria under certain conditions that allows the bacterial body to encapsulate itself within its own secreted polymorphs. The formation of polymicrobial biofilms by the vaginal epithelium plays a critical role in the pathogenesis of BV. Gardnerella is the most capable and virulent biofilm-forming bacterium among the BV-associated pathogenic bacteria (Sun et al., 2013).

**Figure 2:**
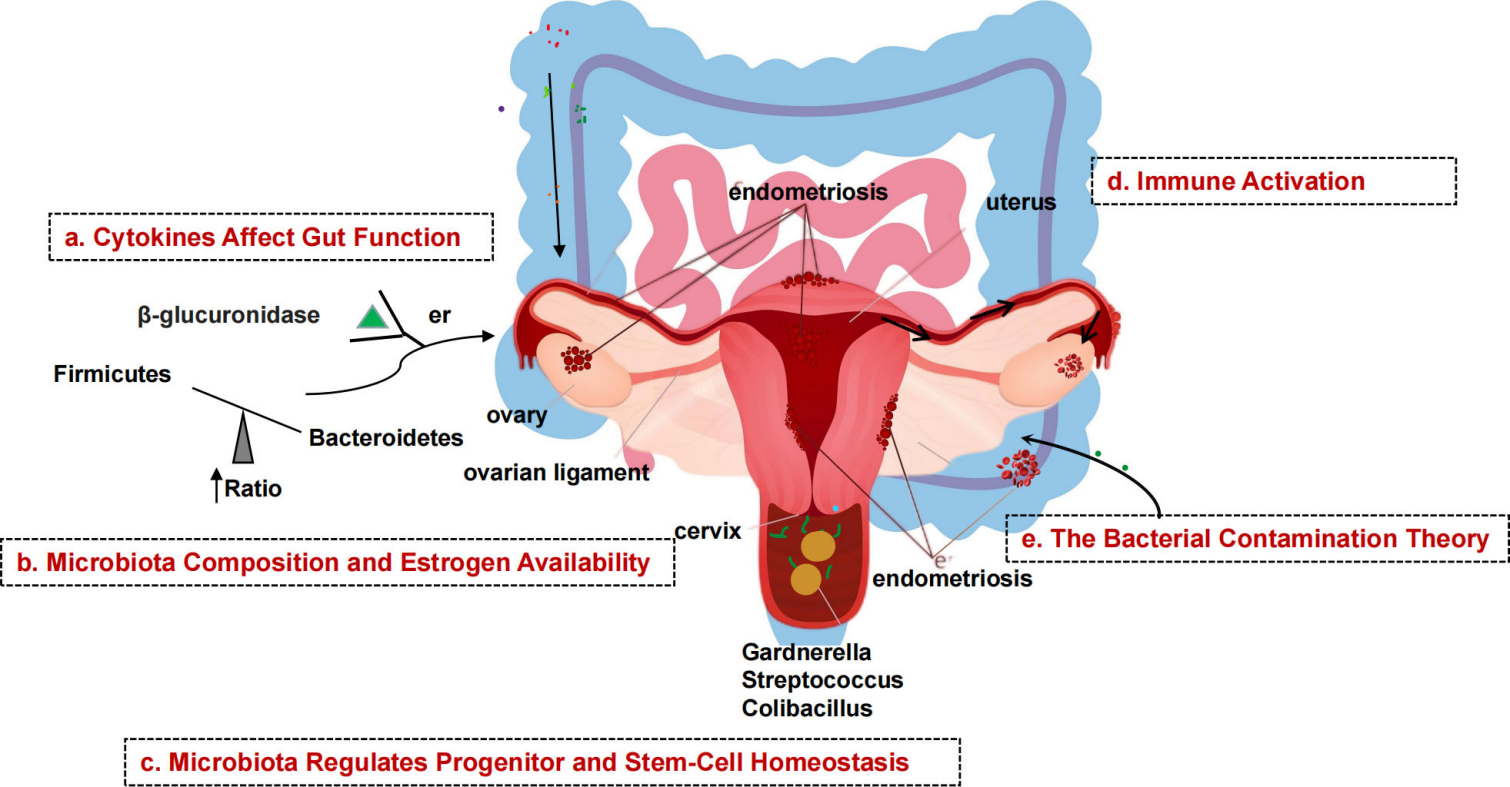
Schematic diagram of the mechanism of microbial involvement in endometriosis. **(A)** Endometriosis causes inflammation of the peritoneum, which suppresses gastric acid production and intestinal motility, allowing gram-negative bacteria to take over. **(B)** β-glucuronidase helps intestinal bacteria metabolize estrogen. β-glucuronidase activates and binds ERs. Gut dysbiosis increases the amount of estrogen that can be delivered to the endometrium *via* the bloodstream. **(C)** Endometrial tissue contains stem cells. Stem cells, which are normally mobile and migrate to the uterus, migrate to ectopic sites *via* the bloodstream, promoting uncontrolled formation of endometrial tissue outside the normal uterine environment, resulting in endometriosis. **(D)** Endometrial fragments that enter the peritoneum during retrograde menstruation produce damage-associated molecular pattern (DAMP) molecules, iron and ROS, activate innate immune cells, and release proinflammatory cytokines and angiogenic growth factors in the peritoneal fluid (PF). Interleukins increase the number of TH17 cells that drive hypervascularization. **(E)** The presence of bacteria in the uterine environment causes endometriosis by refluxing lipopolysaccharides (LPS) into the PF and binding to pattern recognition receptors (PRRs).

Endometriosis (EMs) is a complex gynecologic, noninfectious, inflammatory disease that is often hormone-dependent and frequently associated with pain and infertility, and in severe cases affects the physical and mental health of women of childbearing age (Nezhat et al., 2018). The pathogenesis of EMs is still unclear. In addition to the classic “menstrual blood flow” hypothesis, genetic and immune system dysfunction triggered by oxidative stress have also been linked to the development of Ems (Symons et al., 2018). With the advancement of gene sequencing technology, more and more studies have found that the human microbiota is not only involved in regulating food metabolism and maintaining the integrity of the epithelial lining of the gut, but that its imbalance can also lead to the occurrence of a variety of diseases, such as an imbalanced gut flora that is prone to inflammatory bowel disease and colon cancer, and that the development of EMs may also be related to an imbalance of the flora (Ruan et al., 2020). Dysbiosis of the gut flora disrupts the body’s normal immune function and leads to an increase in pro-inflammatory cytokines, resulting in decreased immune surveillance and altered immune cell function. Over time, this immune dysbiosis can progress to a chronic inflammatory state and create an environment conducive to increased adhesion and angiogenesis, which can drive the development of EMs and promote the vicious cycle of disease onset and progression (Jiang et al., 2021). The microbiota of EMs is associated with decreased Lactobacillus dominance and increased numbers of bacteria associated with bacterial vaginosis and other opportunistic pathogenic bacteria. Possible explanations for abnormalities in EMs flora include theories of bacterial contamination and immune activation, impaired intestinal cytokine function, altered estrogen metabolism and signaling, and abnormal endostasis of progenitor and stem cells. In a pioneering study of EMs induced by intraperitoneal injection of endometrial tissue in mice, the progression of EMs was found to alter the intestinal microbiota. Yuan et al. (Yuan et al., 2018) used intraperitoneal injection to establish a mouse model for EMs and found increased abundance of bifidobacteria in the feces of mice in the advanced group after 42 days, with the ratio of thick-walled bacteria/bacteroidobacteria about twice that of the control group, in which thick-walled bacteria/A recent study by Ata et al. (Ata et al., 2019) compared the composition and abundance of the vaginal, cervical, and intestinal microbiota in patients with EMs III-IV and healthy controls and found that they found differences at the genus level. In the cervical microbiota of women with EMs, they found an increased number of potentially pathogenic species, including Gardnerella, Streptococcus, and Escherichia coli, and a complete absence of the genus Chironomus; in their feces, they showed a significant decrease in the genera Gana, Ciliophora, and a marked dominance of Shigella and Escherichia coli. This suggests that the microbiota of the female gut and reproductive tract may be inextricably linked to the development and progression of EMs (**Figure 2**). This new perspective opens many possibilities for the prevention, diagnosis, and treatment of EMs and is an emerging area of research.

### Neoplastic phase

**Figure 3:**
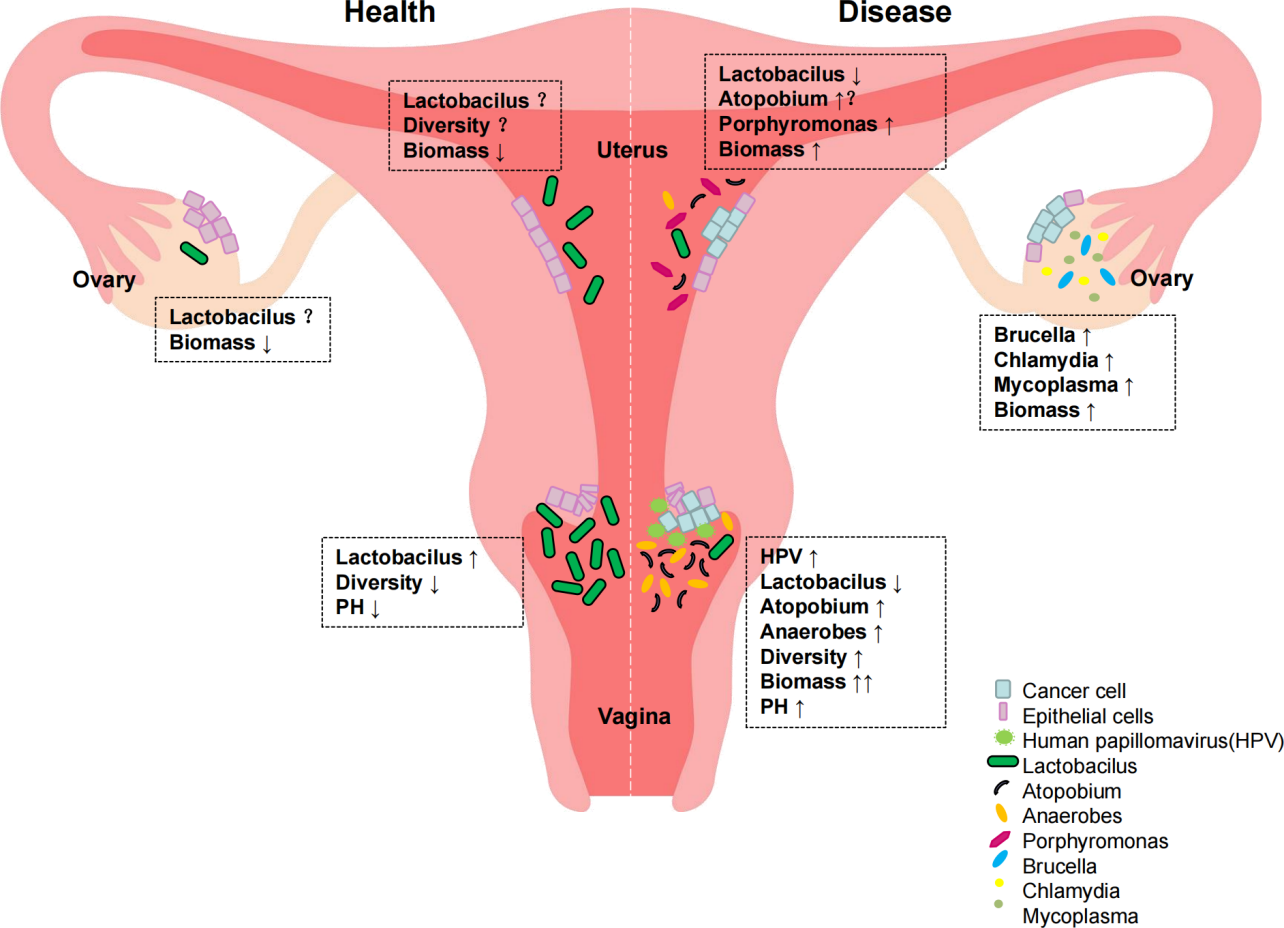
Distribution of reproductive tract colonies in normal women and gynecological cancer patients.

The vaginal microecological balance in women not only plays an important role in infectious diseases of the reproductive tract, but in recent years a possible link between vaginal microecology and gynecological malignancies has also been identified. When dysbiosis occurs, alterations in immune and metabolic signaling may influence cancer characteristics, including chronic inflammation, epithelial barrier dysfunction, alterations in cell growth and apoptosis, genomic instability, angiogenesis, and metabolic dysfunction (Wu et al., 2015). These pathophysiological changes can lead to gynecologic tumors. Emerging evidence suggests that reproductive organ dysfunction and/or certain bacteria may play an active role in the development and/or progression and metastasis of gynecologic malignancies such as cervical, endometrial, and ovarian cancers through direct and indirect mechanisms, including regulation of estrogen metabolism (Łaniewski et al., 2020) (**Figure 3**).

It is well known that cervical cancer is the most common gynecological tumor and that persistent human papillomavirus (HPV) infection, decreased immune function of the body, and changes in the local cervical microenvironment are involved in the development of cervical cancer (Łaniewski et al., 2018). An epidemiologic study found that vaginal microenvironment composition was significantly associated with HPV infection status (P<0.001) (Brotman et al., 2014). The study involved 32 women in whom vaginal secretions were collected twice a week for 16 weeks. The composition of vaginal flora and HPV infection status of each woman were analyzed by second-generation sequencing. The study showed that women with Lactobacillus garciae as the dominant organism had the fastest HPV clearance rate. In addition, women with a vaginal microbiology characterized by low abundance of Lactobacillus garciae and high abundance of Atopobium had the lowest HPV clearance rates. Another study found a significant increase in the diversity of vaginal flora in HPV-infected women (P<0.001) (Gao et al., 2013). In short, a vaginal microecology with Lactobacillus garciae or Lactobacillus inertus as the dominant bacterium is associated with rapid HPV clearance. A decrease in Lactobacillus and an increase in the number of anaerobic bacteria in the vagina are associated with slower HPV clearance, and local cervical microenvironmental changes are strongly associated with the development of cervical cancer.

Gynecologic tumors include endometrial and ovarian cancers in addition to cervical cancer. Studies on these two and vaginal microecology are less well studied and controversial. The development of endometrial cancer is usually associated with excessive estrogen use and environmental factors, including obesity and inflammation (Allen et al., 2008). Available studies have found that these environmental factors are also associated with gut microecology and vaginal microecology (Si et al., 2017). The close association between gut microbiota, estrogen metabolism, and obesity suggests a possible role of microbiota in the etiology of endometrial cancer. A research study on risk factors for endometrial cancer by Kemi M (Doll and Winn, 2019). also confirmed a higher incidence of endometrial cancer in women with dysregulated vaginal microbiota in addition to black and non-Hispanic white women with genital inflammation in the United States. A 2016 study of the potential role of the uterine microbiome in the development of endometrial cancer, which examined the microbiomes of 17 patients with endometrial cancer, 4 with endometrial hyperplasia, and 10 with benign uterine disease in different parts of the genital tract, identified several significantly enriched phyla in samples that were part of the endometrial cancer cohort: Phylum Thicket, Phylum Helicobacter, Phylum Actinomycetes, Phylum Bacteroides, and Anaplasma Phylum. In addition, the presence of Aspergillus and Porphyromonas in association with abnormal vaginal pH (> 4.5) was found to be strongly associated with disease status (Walther-António et al., 2016). Alterations in this microbiota may alter cancer characteristics, including promoting carcinogenesis of endometrial cancer.

Because of its insidious onset and the fact that it is not easily detected, ovarian cancer has the highest mortality rate among the three major gynecologic tumors (Torre et al., 2018). Differences in ethnicity, reproductive history, and oral contraceptive use are all influential factors. In recent years, studies have found that dysregulation of the genital microbiota is associated with ovarian cancer development and is considered a potential biomarker for the disease (Banerjee et al., 2017; Zhou et al., 2019). A 2017 cross-sectional study by Banerjee, S et al. compared ovarian tissue from women with ovarian cancer and healthy participants and identified a unique ovarian microbiome. Furthermore, the microbiota of malignant ovarian tissue had a different microbial profile than healthy ovarian tissue from the same body. Specifically, potentially pathogenic intracellular microorganisms such as Brucella, Mycoplasma, and Chlamydia were found in 60–76% of ovarian tumors. Further studies have demonstrated the presence of several pathogenic viruses and intracellular bacteria in ovarian tissue, including HPV, Chlamydia trachomatis, and cytomegalovirus, which have been identified as biological features of ovarian cancer (Shanmughapriya et al., 2012). Although these preliminary studies have identified various bacteria in ovarian cancer tissue and demonstrated their association with inflammation, the causal relationship between the microbiota and ovarian cancer remains unclear. These microorganisms may trigger carcinogenesis through direct or indirect mechanisms.

The role of reproductive tract flora in gynecologic malignancies and other diseases is receiving increasing attention. Both national and international literature confirm that disturbed vaginal microecology is associated with gynecological malignancies, while a vaginal microecology dominated by lactobacilli is closely related to the health of the organism and may prevent the development of diseases such as tumors. Therefore, restoring or maintaining a healthy vaginal flora has become one of the potential therapeutic approaches in the current treatment of diseases such as cancer (Łaniewski et al., 2020). Probiotics are a general term for a group of active beneficial microorganisms that improve the microecological balance of the host and restore the homeostasis of the microbiome (Falagas et al., 2007). Probiotics have been successfully used in the adjuvant treatment of cancer patients, where they can improve the microecological environment disturbed by tumor treatment and play a role in preventing flora imbalance.

## Frontiers of human microbiomics research

In the last decade, the era of human polyomics has accelerated research in all areas of biology, which is particularly evident in the study of microbial communities and normal flora. In the 18 years since the publication of the first human genome, research on the human microbiome, the Human Gut Microbiome Project Program (HMP) (The Integrative HMP (iHMP) Research Network Consortium, 2019), has evolved from studies of oral and intestinal cultures to molecular profiling of microbial biochemistry in all ecological environments of the human body (Gill et al., 2006; Costello et al., 2009; Wu et al., 2017). Microbial diversity varies in different parts of the body. For example, diversity is generally expected to be greater in the gut, but may be associated with dysbiosis status and risk of adverse events in the female reproductive tract. The HMP is divided into two phases (HMP1 and HMP2). Hmp1 examines the role of microbial communities at various body sites (oral, nasal, vaginal, intestinal, and skin). Hmp2 extends the analysis of host and microbiome biology with three longitudinal cohort studies of typical microbiome-related diseases: Pregnancy and Preterm Birth (Maternal Vaginal Microbiome), Inflammatory Bowel Disease (Gut Microbiome), and Prediabetes (Gut and Nasal Microbiome). In these studies, multiple sequencing methods were used to track many different parts of the human body at different loci, such as changes in microbial community composition, virulence groups, metabolic profiles, host and microbiome gene expression and protein profiles, and host-specific features such as genetic, epigenomic, antibody, and cytokine profiles, as well as other features unique to the study. Key findings from Hmp1: Classification of separate microbiome composition often does not correlate well with host phenotype - a correlation that is often better predicted by the prevalence of microbial molecular functions or personalized strain-specific makeup dressings. This lays the foundation for the development of the second phase of HMP, integrated HMP (iHMP or HMP2). Integrating HMP (iHMP or HMP2) aims to explore host-microbe interactions, including immunological, metabolic, and dynamic molecular activities, to gain a more comprehensive understanding of host-microbe interactions.

**Figure 4:**
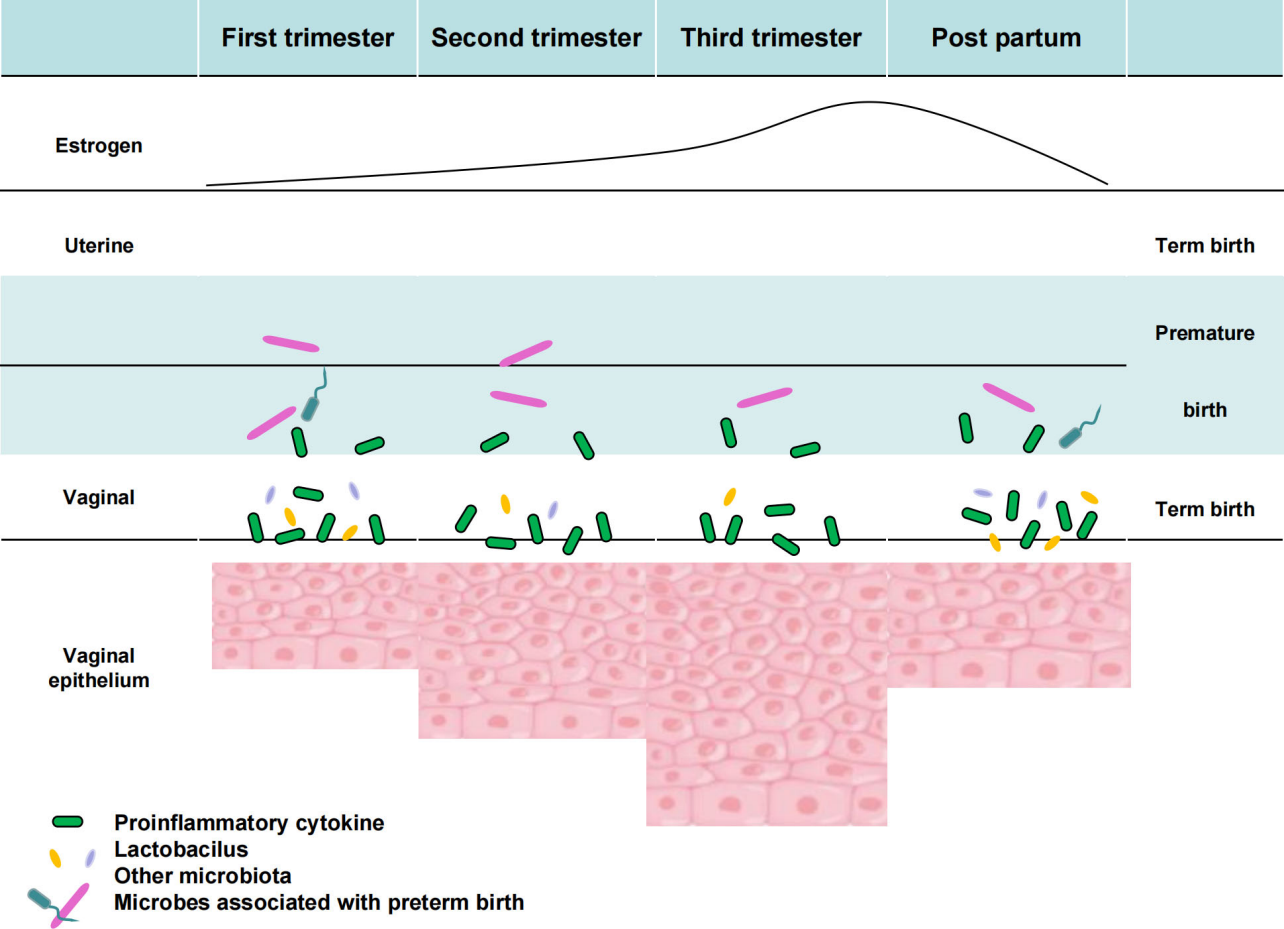
Vaginal flora of preterm pregnancy and its relationship with host factors.

As part of HMP2, the Multiomics Microbiome Study Group-Pregnancy Integration (MOMS-PI) assessed pregnant women’s microbiome to determine its impact on preterm birth risk. The experiment followed 1527 pregnant women longitudinally and collected 206,437 samples, including vaginal, mouth, rectal, skin, and nose swabs, blood, urine, and delivery materials, cord blood, fetal feces and stool, and oral, skin, and rectal swabs. Taxonomic study of the 16S rRNA gene, macrogenome and macrotranscriptome sequencing, cytokine, lipidome, and bacterial genome analyses were performed on these materials. The MOMS-PI team collected 12,039 samples from 597 pregnant women to study preterm labor bacteria and their interactions with the host. As pregnancy proceeds, uterine and vaginal alterations are connected with rising estradiol levels. Early in pregnancy, the uterus is proinflammatory, then anti-inflammatory, and finally proinflammatory soon before childbirth. Specific vaginal microbiome alterations may be linked to premature labor, presumably by microbial translocation to the uterus (**Figure 4**). Studies have shown that differences in vaginal microbial diversity and local vaginal immune environment are associated with an increased risk of preterm birth. In 2018 Son KA studied abnormal vaginal flora colonization in early, mid, and late pregnancy and found that abnormal vaginal flora colonization rates significantly decreased with longer gestation. Abnormal vaginal flora colonization detected in late pregnancy was associated with a significant increase in preterm delivery by 28 weeks of gestation. Among the abnormal vaginal flora isolated in late gestation, the presence of Klebsiella pneumoniae was identified as a significant microorganism associated with preterm birth before 28 weeks of gestation (Son et al., 2018).

And our gut microbiome has a particularly long and detailed history. The gut microbiota is one of the most abundant and well-studied microorganisms. It is known to play an essential role in nutrient absorption and synthesis, maintenance of mucosal integrity, protection from pathogens, and maturation of the immune system (Hooper et al., 2012). In addition to its necessity in maintaining the physiological function of the gastrointestinal tract, it has been found to be an important regulator of many inflammatory and proliferative diseases (West et al., 2015; Pickard et al., 2017; Forbes et al., 2018). In addition, it has been found to influence estrogen metabolism and stem cell homeostasis (Baker et al., 2017). Studies on the human gut microbiome have identified several differences in the gut microbiome and host immune response to disease processes. Studies of the human microbiome have identified novel biological features in both the respective health and disease domains, but including a surprising array of immunological and ecological features of the host microbiome. Studies have found that changes in the microbiota and associated changes in host responses are most pronounced when they originate in the host’s own tissues. Host-microbiome interactions have local and systemic effects. Microbiology as it relates to humans now clearly goes beyond the study of infectious diseases and gastrointestinal disorders and extends into areas that were almost unimaginable just a few decades ago, such as metabolism, oncology, maternal and child health, and central nervous system function. The HMP project has and will continue to reveal many new avenues of research and techniques for future study.

## Summary

This review analyzes the composition and abundance of the microbiota of the female reproductive tract during different physiological and pathological phases (puberty, menstruation, pregnancy, and menopause), as well as the latest research findings in the field of human microbiota. The research presented in this article shows that the vaginal microbiome has different characteristics at different stages of the female reproductive and postreproductive life cycle. Gonadal hormone levels (progesterone and estrogen) were also different at these times. Fluctuations in progesterone and estrogen levels in the female vaginal epithelium and the availability of glycogen may influence the composition and diversity of the vaginal flora at different stages of reproduction. The microorganisms of the reproductive tract influence the health and disease of the body. An imbalance of vaginal bacteria can lead to inflammatory diseases and difficulties in women, including bacterial vaginosis, EMs, and STDs. Abnormal vaginal flora colonization is strongly associated with preterm delivery before 28 weeks of gestation. Understanding the structure and diversity of the reproductive tract microbiome in healthy and pathological conditions is critical for identifying disease risk factors and developing treatments. We believe that the female reproductive tract microbiome is associated with gynecologic malignancies, particularly cervical cancer. However, the host defense mechanisms are still unknown. Further research is needed to determine how microbial communities and/or individual bacterial species in the female reproductive system influence cancer. Future research will use human and physical clinical datasets and *in vitro* models to determine the impact of these microorganisms on homeostasis and disease *in vivo* and how they contribute to gynecologic cancers. Improving studies to better understand host-microbe interactions in the female reproductive system may open new vistas for cancer prevention, treatment, and overall well-being in women.

## Data Availability Statement

The original contributions presented in the study are included in the article/supplementary material. Further inquiries can be directed to the corresponding authors.

## Author Contributions

LS and AQS have performed bibliographic research and drafted the manuscript. All authors contributed to the article and approved the submitted version.

## Funding

This work was supported by the Changning Maternity & Infant Health Hospital Project (2022Y-2), the Postdoctoral Science Foundation of China (2020M681399); the 2021 National Natural Science Foundation of Shanghai Tongji Hospital Incubation Project (TJ202010, TJ2026) and the Medical Research Project of Jiangsu Provincial Health and Health Commission (2019179).

## Conflict of Interest

The authors declare that the research was conducted in the absence of any commercial or financial relationships that could be construed as a potential conflict of interest.

## Publisher’s Note

All claims expressed in this article are solely those of the authors and do not necessarily represent those of their affiliated organizations, or those of the publisher, the editors and the reviewers. Any product that may be evaluated in this article, or claim that may be made by its manufacturer, is not guaranteed or endorsed by the publisher.
